# Can machine learning improve patient selection for cardiac resynchronization therapy?

**DOI:** 10.1371/journal.pone.0222397

**Published:** 2019-10-03

**Authors:** Szu-Yeu Hu, Enrico Santus, Alexander W. Forsyth, Devvrat Malhotra, Josh Haimson, Neal A. Chatterjee, Daniel B. Kramer, Regina Barzilay, James A. Tulsky, Charlotta Lindvall

**Affiliations:** 1 Department of Radiology, Masachusetts General Hospital, Boston, Massachusetts, United States of America; 2 Department of Electrical Engineering and Computer Science, CSAIL, MIT, Cambridge, Massachusetts, United States of America; 3 Department of Health Policy and Management, Harvard School of Public Health, Boston, Massachusetts, United States of America; 4 Division of Cardiology, Department of Medicine, University of Washington, Seattle, Washington, United States of America; 5 Richard A. and Susan F. Smith Center for Outcomes Research, Division of Cardiology, Beth Israel Deaconess Medical Center, Boston, Massachusetts, United States of America; 6 Department of Psychosocial Oncology and Palliative Care, Dana-Farber Cancer Institute, Boston, Massachusetts, United States of America; 7 Division of Palliative Medicine, Department of Medicine, Brigham and Women’s Hospital, Boston, Massachusetts, United States of America; University Hospital *Paolo Giaccone*, ITALY

## Abstract

**Rationale:**

Multiple clinical trials support the effectiveness of cardiac resynchronization therapy (CRT); however, optimal patient selection remains challenging due to substantial treatment heterogeneity among patients who meet the clinical practice guidelines.

**Objective:**

To apply machine learning to create an algorithm that predicts CRT outcome using electronic health record (EHR) data avaible before the procedure.

**Methods and results:**

We applied machine learning and natural language processing to the EHR of 990 patients who received CRT at two academic hospitals between 2004–2015. The primary outcome was reduced CRT benefit, defined as <0% improvement in left ventricular ejection fraction (LVEF) 6–18 months post-procedure or death by 18 months. Data regarding demographics, laboratory values, medications, clinical characteristics, and past health services utilization were extracted from the EHR available before the CRT procedure. Bigrams (*i*.*e*., two-word sequences) were also extracted from the clinical notes using natural language processing. Patients accrued on average 75 clinical notes (SD, 29) before the procedure including data not captured anywhere else in the EHR. A machine learning model was built using 80% of the patient sample (training and validation dataset), and tested on a held-out 20% patient sample (test dataset). Among 990 patients receiving CRT the mean age was 71.6 (SD, 11.8), 78.1% were male, 87.2% non-Hispanic white, and the mean baseline LVEF was 24.8% (SD, 7.69). Out of 990 patients, 403 (40.7%) were identified as having a reduced benefit from the CRT device (<0% LVEF improvement in 25.2%, death by 18 months in 15.6%). The final model identified 26% of these patients at a positive predictive value of 79% (model performance: F_β_ (β = 0.1): 77%; recall 0.26; precision 0.79; accuracy 0.65).

**Conclusions:**

A machine learning model that leveraged readily available EHR data and clinical notes identified a subset of CRT patients who may not benefit from CRT before the procedure.

## Introduction

Cardiac resynchronization therapy (CRT) is an established therapy for patients with medically refractory systolic heart failure and left ventricular dyssynchrony[1–5]. Improvement of left ventricular ejection fraction (LVEF) following CRT implant is associated with a reduction in heart failure hospitalizations and improved survival. Despite these established benefits, at least one-third of CRT patients do not experience an improvement in LVEF 6–18 months following the procedure[6]. Another subgroup of patients die from heart failure or other comorbidities before the effectiveness of CRT can be measured. These patients are exposed to procedural risks and cost that may be preventable with improved patient selection [7].

Current consensus guidelines regarding selection for CRT implantation focus on a limited set of patient characteristics including NYHA functional class, LVEF, QRS duration, type of bundle branch block, etiology of cardiomyopathy and atrial rhythm (sinus, atrial fibrillation). There is also a subjective assessment of ‘general health status’, which broadly reflects the patient’s other comorbidities, although guidance and details regarding making this assessment are lacking[8,9]. While several clinical factors have been associated with reduced benefit of CRT, no prediction models are routinely used in clinical practice to support optimal patient selection[10]. Recent advances in computer science together with the use of Electronic Health Records (EHR) support ‘machine learning’ algorithms that iteratively learn from complex longitudinal EHR data[11,12]. Machine learning can integrate with natural language processing and thereby make use of both the structured (*e*.*g*., lab values, medications) and unstructred free text (*e*.*g*., clinical notes) EHR data. Machine learning algorithms that process thousands or even millions of variables hold the promise to improve on both the precision and usability of existing prediction models.

We built and tested a machine learning model that incorporates both structured and unstructured data from the EHR, and applied it to a retrospective cohort of CRT recipients at two academic hospitals. We aimed to test the effectiveness of machine learning and natural language processing to predict clinical outcome after CRT.

## Methods

### Data source

Our primary data source was the Partners HealthCare Research Patient Data Registry[13]. Research Patient Data Registry gathers data from multiple hospital electronic record systems at Partners HealthCare, a large network in Massachusetts, and includes greater than 20 years of data from 4.6 million patients. The database contains over 227 million encounters, 193 million billing diagnoses, 105 million medications, 200 million procedures, 852 million lab values, and over 5 million unstructured clinical notes, which include outpatient visit notes, inpatient admission and consultation notes, cardiology reports, and others. Patient-level data are available for research projects after peer-reviewed proposal review. This study was approved by the Partners Human Research Committee Prior to analyzing the dataset, Partners Human Research Committee waived the requirement for informed consent because we used retrospective EHR data and most patients included in the study had died.

### Study population

Patients undergoing CRT implantation between January 2004 and December 2015 at Massachusetts General Hospital and Brigham and Women’s Hospital were eligible. Cases were identified using CRT procedure codes ICD9 00.50, ICD9 00.51, CPT 33224, CPT 33225 or CPT 33226. We included patients who received an initial CRT device, either CRT pacemaker (CRT-P) or CRT with implantable cardioverter defibrillator (CRT-D). We excluded from analysis patients who did not have a baseline measurement of LVEF within 60 days of the procedure, follow-up LVEF measurement between 6 months to 18 months post-procedure and were alive at 18 months, or who received a CRT within 18 months of the end of the dataset’s time window. We did not require follow-up LVEF on patients who died within 18 months of CRT device placement.

### Primary outcome

The primary outcome was defined as <0% improvement in LVEF 6–18 months following CRT implantation or death within 18 months of CRT. The baseline LVEF was the value measured closest to the procedure date and within 60 days before CRT. All LVEF values were extracted using regular expression natural language processing from echocardiogram or cardiac catherization reports. We manually confirmed that the extracted values were correct by viewing the report using Localturk, an open source implementation of Amazon’s Mechanical Turk API[14]. If multiple LVEF measurements were available at each time point, the measurement obtained closest to the procedure date was used as the baseline LVEF and the measurement obtained closest to 12 months following the procedure as the follow up LVEF.

Patient death within 18 months after CRT was considered reduced CRT benefit regardless of LVEF. In fact, we did not require follow-up LVEF for patients who died because of missing data. Post-procedure LVEF was often not measured before death and therefore, we do not report change in LVEF for these patients. Date of death was obtained using the social security death index available in Research Patient Data Registry.

### Study variables

Research Patient Data Registry contains both structured and unstructured data.

#### Structured data

Table 1 details the longitudinal EHR data that was used to build the feature vectors. In addition to **demographic information** (*e*.*g*., sex, age), we used structured EHR data available before the CRT procedure including billing codes, encounter information (*i*.*e*., visit type, length of stay), laboratory reports (*e*.*g*., lab values), medication lists and cardiology reports (*e*.*g*., LVEF, QRS).

**Table 1:** Structured data vectors utilized in machine learning algorithms.

Data source	Variables
Demographics	Sex; age at implant; race
Billing codes	ICD-9-CM
Encounter data	Visit type; length of stay; number of diagnosis codes; inpatient ratio; average length of stay; average number of diagnosis codes
Laboratory reports	Lab values; most recent and trend
Medication list	Medication and drug class
Cardiology reports	LVEF; QRS; LBBB; Sinus rhythm; most recent and trend

The Clinical Classifications Software (CCS) developed by the Healthcare Cost and Utilization Project (HCUP) was used to transform each individual International Classification of Diseases, Ninth Revision, Clinical Modification (ICD-9 CM) code into a hierarchy of disease[15]. For example, the ICD-9 CM diagnosis code for Endocardial fibroelastosis is 425.3, but using CCS we converted this into the code 7.2.2, where 7 represents “Disease of the Circulatory System,” 7.2 represents “Heart Disease,” and 7.2.2 represents “Cardiomyopathy.” A one-hot encoding was created for each level in the hierarchy and they were then concatenated into a single vector, allowing us to simultaneously represent a disease like Endocardial fibroelastosis as “Disease of the Circulatory System,” “Heart Disease,” “Cardiomyopathy,” and “Endocardial fibroelastosis.” Once we had a vector for each diagnosis in a patient’s medical history, we summed these vectors and normalized their values to binary numbers, where 1 represented any history of disease at that level of the hierarchy and 0 represented no history of disease at that level of the hierarchy. The resulting **diagnosis vector** then represented each patient’s diagnosis history.

Similarly, the SNOMED clinical terminology was used to transform each individual medication into a hierarchy of medications by creating one-hot encodings for the RxNorm medication classes[16]. The resulting **medication vector** then represented each patient’s medication history.

The **encounter vector** was produced using the last five patient visits prior to the device implantation. For each encounter, the visit type (0 for outpatient and 1 for inpatient), the length of stay, and the number of additional diagnoses were extracted, resulting in 15 different features (5 encounters x 3 variables). We also included the summaries of these features, encoding them as the inpatient ratio, the average length of stay, and the average number of diagnoses code.

We also created a **laboratory vector** to represent the laboratory results throughout the patient’s history. For each available lab test, the laboratory vector included total number of entities, total counts of abnormal events (too high or too low), and the most recent lab testing values.

The variables used in the CRT clinical practice guidelines including LVEF, QRS, LBBB pattern, sinus rhythm, and NYHA class were not available as structured data fields in Research Patient Data Registry. We therefore encoded them into a **cardiology vector**, after extracting them via natural language processing regular expressions followed by manual review by physician (CL) from electrocardiogram reports (QRS, LBBB, sinus rhythm), echocardiogram or cardiac catherization reports (LVEF), and clinical notes (NYHA class).

#### Unstructured data

To assess whether unstructured clinical texts could contribute to increase the prediction accuracy, we first used natural language processing techniques to explore the relationship between words in the clinical notes and the primary outcome. After applying minor preprocessing steps to reduce the noise (i.e. words were lowercased; stop words, numbers, special characters and low-frequency bigrams were removed (frequency<5), we calculated the association between the bigrams (*i*.*e*., two-word sequences) and the primary outcome with local mutual information (LMI) [17]. LMI measures the interdependency between words and labels, while also adjusting for the word frequency to avoid bias toward rare events. For a given bigram (*w*) and a label (*l*), LMI is formulated as:

$$LMI(w,l)=p(w,l)\times log(\frac{p(l|w)}{p(l)})$$

where $p(lw)=\frac{count(w,l)}{count(w)},p(l)=\frac{count(l)}{\left|D\right|},p(w,l)=\frac{count(w,l)}{\left|D\right|}$. *|D|* is the number of the occurences of the bigrams in the dataset.

We encode each clinical note as a weighted average of the word embeddings[18] which are vectors representing the semantics of the words that appear in the note. We train a word2vec continuous bag-of-words model on clinical notes from Research Patient Data Registry, using Python packaged with Gensim for word embeddings[19]. We did not use pre-trained word embeddings or train on a public dataset because we wanted medical specific word embeddings. We estimated the weight of each word in the note using the word frequency-inverse document frequency (tf-idf), which keeps track of the word relevance in the document. Word tf-idf weights a word’s term frequency by a factor inversely proportional to the number of documents in the entire corpus in which that word appears, adjusting for the fact that some words are more frequent than others.

### Machine learning

**Fig 1:**
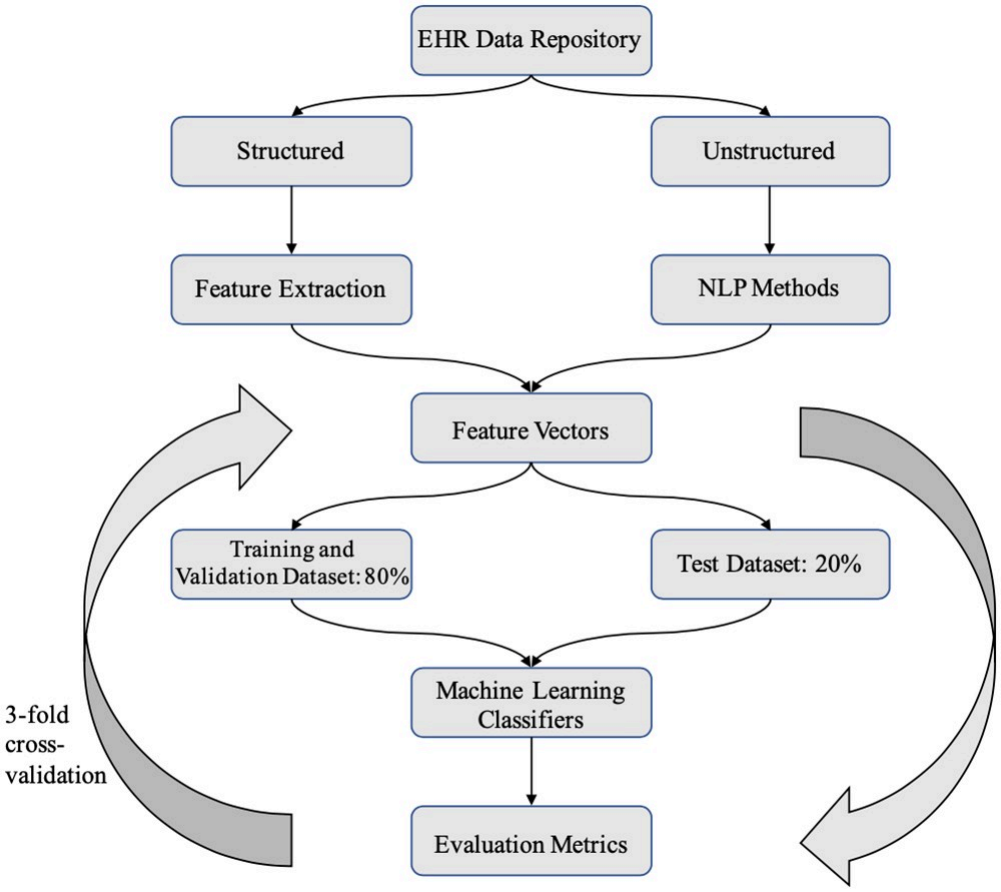
Overview of computational method.

Several machine learning classifiers, including logistic regression, support vector machine, random forest and gradient boosting classifiers were trained to predict reduced benefit of CRT using Python, scikit-learn[20] and XGBoost package[21]. The patient sample was divided into a training and validation (80% sample) dataset and a test (20% sample) dataset. For each classifier, a hyperparameter search was performed and the model with the best F_β_ score on 3-cross validations was selected. Once the final model had been selected, it was run on the held-out 20% test dataset to obtain final performance metrics. The test dataset was not used at any step in model building. The overview of the pipeline is shown in Fig 1.

### Statistical analysis

Baseline characteristics were described using proportions, means +/- SD, and medians as appropriate. The prediction models were evaluated by precision (positive predicted value), recall (sensitivity), and a weighted F-measure, F_β_, established performance metrics in machine learning. True Positives (TP) were defined as the number of CRT correctly classified as ineffective (*i*.*e*., reduced benefit measured by change in LVEF or death); False Negatives (FN) as the number CRT treatments classified as effective that were actually ineffective; False Positives (FP) as the number of CRT treatments classified as ineffective that were actually effective.

$$\begin{array}{lll} precision=\frac{TP}{TP+FP}, & recall=\frac{TP}{TP+FN}, & accuracy=\frac{TP+TN}{TP+TN+FP+FN} \end{array}$$

To reduce the number of false positive, we trained the model to favor precision over recall. To this end, a weighted F-measure (F_β_) was used to weigh precision higher than recall, beta = 0.1.

$$F_{\beta }=(1+\beta^{2})\times \frac{precision\times recall}{\beta^{2}\times precision+recall}$$

### Reproducible research statement

The code used in the paper is publicly available at https://github.com/lindvalllab/MLCRT.

## Results

### Patient characteristics

We identified 2,641 eligible CRT recipients using procedure billing codes. 1,651 of the 2,641 patients were missing either a baseline or follow up LVEF measurement in the acceptable time ranges, or received CRT with 18 months of the end of the dataset’s time window, and were thus excluded from further study. The final study sample included 990 patients. We manually confirmed that these patients received CRT for the first time by reviewing their procedure reports.

The baseline demographic and clinical characteristics of the study sample are presented in Table 2. The mean age of CRT device recipients was 71.6 years old, with the majority of individuals being male (78.1%) and non-Hispanic white (87.2%). The most common comorbidities included non-ischemic heart failure (80.2%), coronary artery disease (74.6%), ventricular arrhythmia (60.3%) and atrial fibrillation (50.8%). 37.8% of implant recipients had left bundle branch block. Baseline diagnostic studies showed mean LVEF of 24.8%, creatinine of 1.69, sodium of 137.4 and hemoglobin of 12.4. A majority of patients were on beta-blockers (92.3%), and angiotensin converting enzyme inhibitors or angiotensin receptor blockers therapy (77.3%).

**Table 2:** Baseline patient characteristics. All values were obtained prior to CRT implant.

	All (n = 990)	Reduced CRT benefit (n = 403)	CRT non-progressor (n = 587)	*p*-value
Age, mean (SD), y	71.6 (11.8)	72.2	71.2	0.21
Female, %	21.9	17.6	24.9	<0.001
Non-Hispanic white, %	87.2	85.1	88.6	<0.001
Medical history, %				
Non-ischemic heart failure	80.2	71.2	86.4	<0.001
NYHA class II or III	94.9	89.7	96.9	<0.001
Coronary artery disease	74.6	80.7	71.2	<0.001
Left bundle branch block	37.8	24.8	46.8	<0.001
Ventricular arrhythmia	60.3	62.3	58.9	<0.001
Atrial fibrillation	50.8	57.6	46.2	<0.001
Diabetes mellitus	32.7	40.0	27.8	<0.001
Diagnostic studies, mean (SD)				
LVEF, (%)	24.8 (7.69)	28.1	23.9	<0.001
QRS duration, (ms)	153.3 (33.3)	152.1	154.0	<0.001
Resting heart rate, (bpm)	82.5 (38.9)	82.8	82.2	0.44
Creatinine, (mg/dL)	1.69 (1.17)	1.63	1.76	0.35
Sodium, (mEq/L)	137.4 (3.9)	137.4	137.4	0.93
Hemoglobin, (mg/dL)	12.4 (2.0)	12.0	12.8	<0.001
Medications, %				
Beta-blocker	92.3	92.3	92.3	0.98
ACE/ARB	89.4	88.8	89.8	0.53

### Primary outcome

Out of 990 patients, 403 (40.7%) were identified as having a reduced benefit from the CRT device: 249 patients died within 18 months of device implantation (25.2%) and 154 had <0% improvement in LVEF post-procedure (15.6%). 587 patients were non-progressors, defined as ≥0% improvement in LVEF post-procedure (59.3%). Thus, physicians selected patients for CRT implantation who subsequently responded to therapy with an accuracy of 0.59. Recall (*i*.*e*., sensitivity) and precision (*i*.*e*., positive predictive value) could not be calculated for the empiric physician-selected CRT strategy because all patients who were included in this study received CRT. The baseline demographic and clinical characteristics of patients stratified by the primary outcome are presented in Table 2.

### Model performance

**Fig 2:**
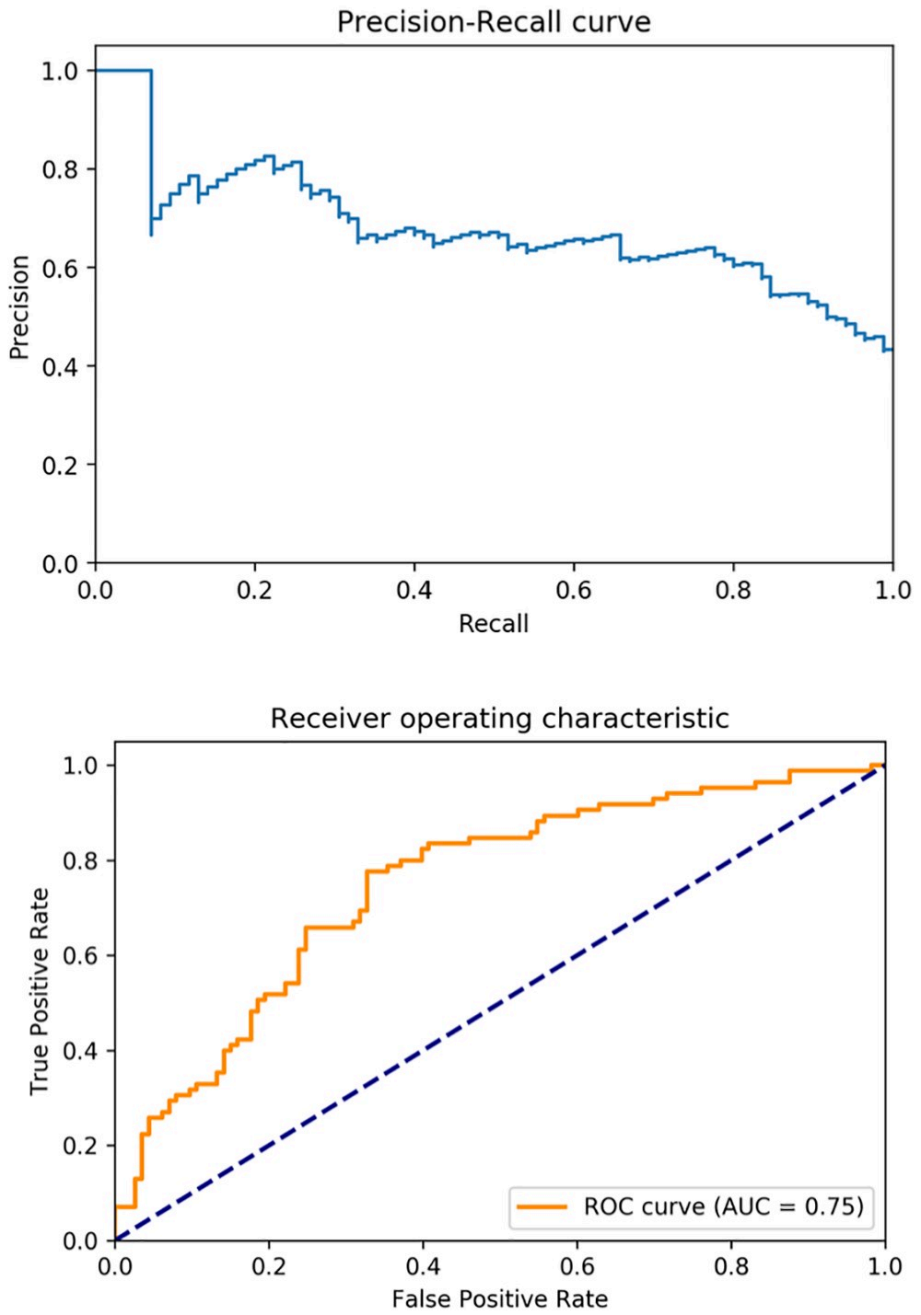
Precision-recall curve and ROC curve of the final model.

**Fig 3:**
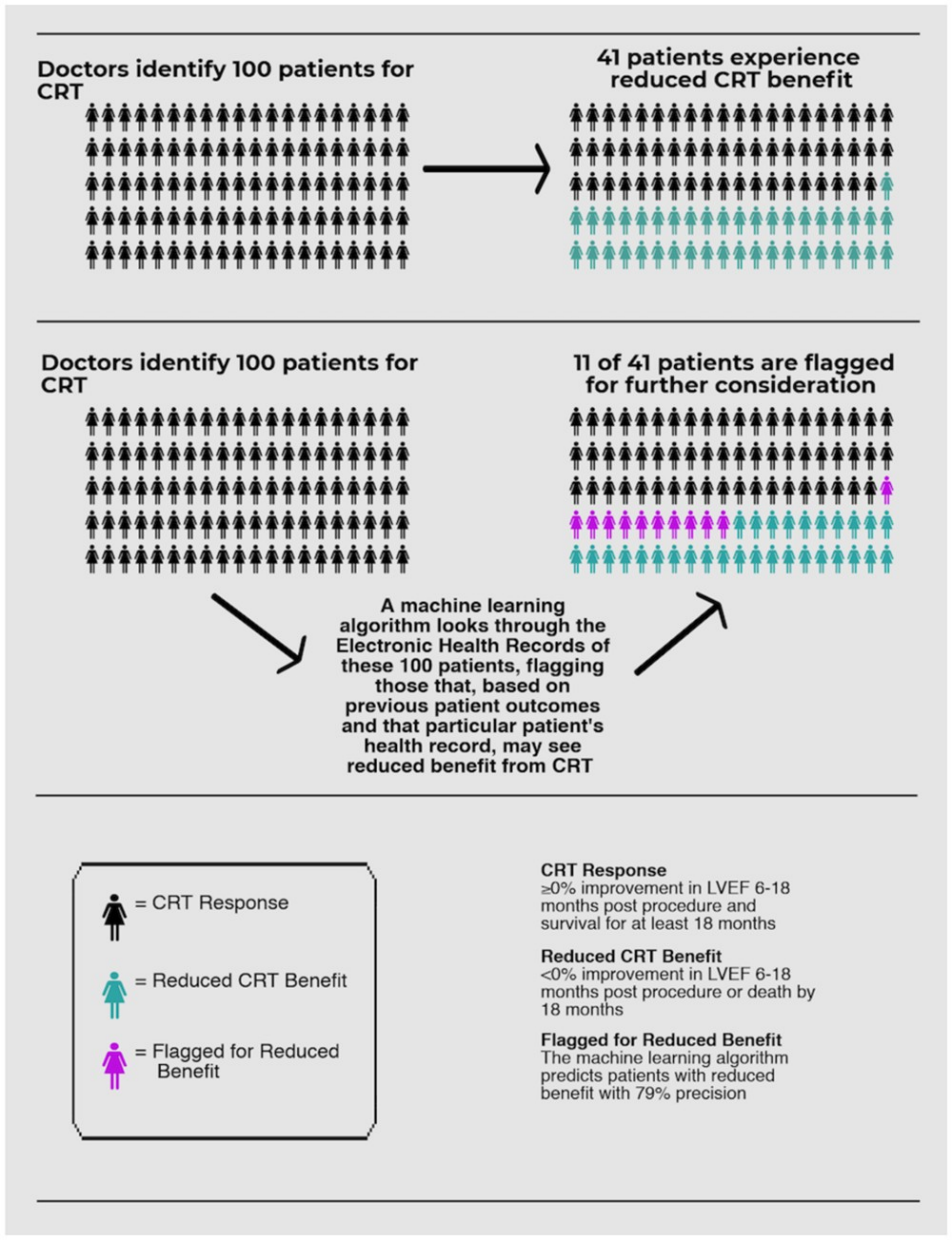
Illustration of envisioned clinical utilization of machine learning prediction.

In our experiment, the gradient boosting classifier gave the best F_β_ score in the 3-fold cross-validation, and it was adopted to determine the performance on a held-out sample of 200 patients from the 990 total sample. All results presented here were obtained from running the final machine learning model on the test dataset. Our final machine learning model, which utilized both structured features and natural language processing bigrams from the clinical notes, identified 26% of the patient population who experienced reduced CRT benefit with a precision of 79% (F_β_ (β = 0.1): 77%; recall 0.26; precision 0.79; accuracy 0.65). As expected, our model has a significantly higher precision compared to the recall since we were optimizing over F with β = 0.1. This beta value can be tailored according to different clinical needs. Fig 2 shows the final model performance with the precision-recall curve and receiver operating characteristic (ROC) curve, both of which illustrates the trade-off between false positives and false negatives under different discriminative thresholds. Table 3 lists the top LMI-ranked bigrams related to the reduced benefits: they are mostly clinically relevant phrases describing pathophysiology (*e*.*g*., *volume overload*, *ventricular tachycardia*), organ dysfunction (*e*.*g*., *renal failure*) or treatments (*e*.*g*., *warfarin sodium*). The pictograph in Fig 3 demonstrates frequencies of patients predicted to have reduced CRT benefit by the model and their actual clinical outcome.

**Table 3:** Examples of bigrams extracted from clinical notes, listed in order of relative importance ranked by local mutual information.

Bigram
heart failure
aortic valve
renal function
renal failure
volume overload
60 tablet
coronary artery
warfarin sodium
congestive heart
take tablet
renal insufficiency
lung cancer
followed dr
artery disease
ventricular tachycardia
mean gradient
chf exacerbation
phone call
chf ef
allopurinol 100
heart failure
aortic valve
renal function
renal failure
volume overload
60 tablet
coronary artery
warfarin sodium

## Discussion

Machine learning applied to EHR data identified a subgroup of patients who were unlikely to benefit from CRT. We built a machine learning model that utilized structured data (*e*.*g*., labs, medications, billing codes) and nondiscrete text data from clinical notes (*i*.*e*., bigrams) available in the EHR before the CRT procedure. The model predicted the binary outcome ‘reduced CRT benefit’ for each patients and identified 26% of patients who experienced <0% improvement in LVEF or died before 18 months with a positive predictive value of 79%. Although our initial model requires optimization and training on additional dataset to improve the performance, further model building may yield machine learning methods suitable to be deployed in routine clinical care to augment shared decision making prior to CRT placement.

Accurate patient selection is important to minimize morbidity and mortality related to the device, and to control healthcare costs[7]. It will only become more important as the population of heart failure patients continues to grow. However, more than a decade of work has shown that it is not easy to identify new predictors of CRT response[6,22–24]. For example, a multicenter prospective study, Predictors of Response to CRT Trial (PROSPECT), found that no single echocardiographic measure of dyssynchrony could be recommended to improve patient selection for CRT[10]. The amount of data available in the EHR is massive, and is rapidly expanding. Analysis of these data using methods from computer science can allow for discovery of complex patterns that are clinically important, but difficult for the human mind to identify. Machine learning does not require prior assumptions about causative variables and allows for an exploration of all available data for non-linear patterns[12]. In our study, we utilized both structured EHR data and unstructured clinical text data. Patients with chronic illnesses, such as heart failure, often accrue hundreds of clinical notes over their illness course. These notes contain data from clinical visits that is interpreted and compressed by clinicians, emphasizing important events and assessments. Machine learning with natural language processing provides novel opportunities to utilize this untapped source of data in predictive modeling.

### Clinical implications

Machine learning that utilizes EHR data has the potential to support personalized clinical decisions for patients. Validated models that run in the background of the EHR could enable recognition of numerous marginal risk factors, which by themselves may not reach significance, but put together can provide a more individualized risk prediction. This is clinically important because these methods could augment current guidelines-based approach. Clinicians often encounter patients with demographic and clinical characteristics that differ from the patients who participated in the studies that formed the evidence basis for the guidelines[25,26]. Thousands of CRT procedures are performed in the United States alone every month and so opportunities for refined decision support tools should be pursued.

Though these methods are novel in this specific context, they are not unprecedented in cardiology. For example, a study that analyzed clinical data of 378,256 patients from United Kingdom family practices showed that a machine learning model successfully predicted 7.4% more cases of cardiovascular events compared to the American College of Cardiology guidelines[27]. Machine learning has also been shown to better predict 5-year all-cause mortality for patients who underwent coronary computed tomographic angiography for evaluation of suspected coronary artery disease compared to clinical or coronary computed tomographic angiography metrics alone[28]. Additionally, a recent study utilized data from the COMPANION trial to build a random forest model that predicted all-cause mortality or heart failure hospitalization in CRT pateients[29]. We expand upon that work by building a model that utilized both structured and clinical text data from the EHR.

### Limitations

Our study provides proof of concept, yet several limitations hinder its current applicability in clinical care. First, the model was designed, validated and tested on retrospective data from patients who received CRT implants. Our model has not been validated in a setting where physicians are trying to determine CRT implantation in heart failure patients. Second, our study did not distinguish between the type of CRT implant: CRT-D and CRT-P. Past studies have shown a mortality difference between the two groups. The vast majority of CRT implants in the United States, including Partners HealthCare, are CRT-D[30]. Third, our model used EHR data from one academic healthcare system. There are institutional and provider differences in quantity and quality of EHR data. Differences in geographic and institutional patient populations may make our machine learning model perform poorly in other settings. Even if our model could be applied to other healthcare systems that would require modifications of the code to work on data from different EHR systems. Fourth, predictors are not causes; caveats of epidemiological research including confusing correlation with causation still apply[31]. Also, although we included structured and unstructured EHR data, additional parsing from imaging[32], electrocardiography (*e*.*g*., QRS index[33,34]), and echocardiogram reports (*e*.*g*., left ventricular end diastolic dimension) may have improved the model performance. A persistent source of uneasiness with machine learning models is that they achieve results in a “black-box” manner[35]. Machine learning models often lack explanatory power and the pathophysiological relation between patient-level variable and CRT response remain unclear. Significant efforts are underway to develop interpretable machine learning models[36].

In summary, machine learning identified a subset of CRT patients who may not benefit from CRT. Machine learning models that utilize big EHR data including clinical text data may be used in the future to support patient selection and shared decision making.
